# Pre-diagnostic metabolite concentrations and prostate cancer risk in 1077 cases and 1077 matched controls in the European Prospective Investigation into Cancer and Nutrition

**DOI:** 10.1186/s12916-017-0885-6

**Published:** 2017-07-05

**Authors:** Julie A. Schmidt, Georgina K. Fensom, Sabina Rinaldi, Augustin Scalbert, Paul N. Appleby, David Achaintre, Audrey Gicquiau, Marc J. Gunter, Pietro Ferrari, Rudolf Kaaks, Tilman Kühn, Anna Floegel, Heiner Boeing, Antonia Trichopoulou, Pagona Lagiou, Eleutherios Anifantis, Claudia Agnoli, Domenico Palli, Morena Trevisan, Rosario Tumino, H. Bas Bueno-de-Mesquita, Antonio Agudo, Nerea Larrañaga, Daniel Redondo-Sánchez, Aurelio Barricarte, José Maria Huerta, J. Ramón Quirós, Nick Wareham, Kay-Tee Khaw, Aurora Perez-Cornago, Mattias Johansson, Amanda J. Cross, Konstantinos K. Tsilidis, Elio Riboli, Timothy J. Key, Ruth C. Travis

**Affiliations:** 1 Cancer Epidemiology Unit, Nuffield Department of Population Health, University of Oxford, Oxford, OX3 7LF UK; 2 International Agency for Research on Cancer, 69372 Lyon, CEDEX 08 France; 3 Department of Epidemiology and Biostatistics, School of Public Health, Imperial College London, London, W2 1PG UK; 4 Division of Cancer Epidemiology, German Cancer Research Center (DKFZ), Foundation under Public Law, DE-69120 Heidelberg, Germany; 5 Department of Epidemiology, German Institute of Human Nutrition (DIfE) Potsdam-Rehbrücke, DE-14558 Nuthetal, Germany; 6 Hellenic Health Foundation, GR-11527 Athens, Greece; 7 WHO Collaborating Center for Nutrition and Health, Unit of Nutritional Epidemiology and Nutrition in Public Health, Department of Hygiene, Epidemiology and Medical Statistics, School of Medicine, National and Kapodistrian University of Athens, GR-11527 Athens, Greece; 8 Department of Epidemiology, Harvard T. H. Chan School of Public Health, 02115 Boston, Massachusetts USA; 9 Epidemiology and Prevention Unit, Fondazione IRCCS Istituto Nazionale dei Tumori, Via Venezian, 1, 20133 Milano, Italy; 10 Cancer Risk Factors and Life-Style Epidemiology Unit, Cancer Research and Prevention Institute – ISPO, 50134 Florence, Italy; 11 Cancer Epidemiology Unit-CERMS, Department of Medical Sciences, University of Turin, 10126 Turin, Italy; 12 CPO-Piemonte, 10126 Turin, Italy; 13 Cancer Registry and Histopathology Unit, “Civic-M.P.Arezzo” Hospital, ASP 97100 Ragusa, Italy; 14 Department for Determinants of Chronic Diseases (DCD), National Institute for Public Health and the Environment (RIVM), 3720 BA Bilthoven, The Netherlands; 15 Unit of Nutrition and Cancer, Cancer Epidemiology Research Program, Catalan Institute of Oncology-IDIBELL, 08908 L’Hospitalet de Llobregat Barcelona, Spain; 16 Public Health Division of Gipuzkoa, Regional Government of the Basque Country, 20014 Donostia-San Sebastián, Spain; 17 CIBER of Epidemiology and Public Health (CIBERESP), Madrid, Spain; 18 Escuela Andaluza de Salud Pública, Instituto de Investigación Biosanitaria ibs.GRANADA, Hospitales Universitarios de Granada/Universidad de Granada, 18012 Granada, Spain; 19 Navarra Public Health Institute, 31003 Pamplona, Spain; 20 Navarra Institute for Health Research (IdiSNA) Pamplona, Pamplona, Spain; 21 Department of Epidemiology, Murcia Regional Health Council, IMIB-Arrixaca, 30003 Murcia, Spain; 22 Public Health Directorate, 33006 Asturias, Spain; 23 MRC Epidemiology Unit, University of Cambridge, CB2 0SR Cambridge, UK; 24 School of Clinical Medicine, University of Cambridge, CB2 2QQ Cambridge, UK; 25 Department of Hygiene and Epidemiology, School of Medicine, University of Ioannina, 45110 Ioannina, Greece

**Keywords:** Acylcarnitines, Amino acids, Biogenic amines, European Prospective Investigation into Cancer and Nutrition (EPIC), Glycerophospholipids, Hexose, Mass spectrometry, Metabolomics, Prospective study, Prostate cancer risk, Sphingolipids

## Abstract

**Background:**

Little is known about how pre-diagnostic metabolites in blood relate to risk of prostate cancer. We aimed to investigate the prospective association between plasma metabolite concentrations and risk of prostate cancer overall, and by time to diagnosis and tumour characteristics, and risk of death from prostate cancer.

**Methods:**

In a case-control study nested in the European Prospective Investigation into Cancer and Nutrition, pre-diagnostic plasma concentrations of 122 metabolites (including acylcarnitines, amino acids, biogenic amines, glycerophospholipids, hexose and sphingolipids) were measured using targeted mass spectrometry (Absolute*IDQ* p180 Kit) and compared between 1077 prostate cancer cases and 1077 matched controls. Risk of prostate cancer associated with metabolite concentrations was estimated by multi-variable conditional logistic regression, and multiple testing was accounted for by using a false discovery rate controlling procedure.

**Results:**

Seven metabolite concentrations, i.e. acylcarnitine C18:1, amino acids citrulline and *trans*-4-hydroxyproline, glycerophospholipids PC aa C28:1, PC ae C30:0 and PC ae C30:2, and sphingolipid SM (OH) C14:1, were associated with prostate cancer (*p* < 0.05), but none of the associations were statistically significant after controlling for multiple testing. Citrulline was associated with a decreased risk of prostate cancer (odds ratio (OR_1SD_) = 0.73; 95% confidence interval (CI) 0.62–0.86; *p*_trend_ = 0.0002) in the first 5 years of follow-up after taking multiple testing into account, but not after longer follow-up; results for other metabolites did not vary by time to diagnosis. After controlling for multiple testing, 12 glycerophospholipids were inversely associated with advanced stage disease, with risk reduction up to 46% per standard deviation increase in concentration (OR_1SD_ = 0.54; 95% CI 0.40–0.72; *p*_trend_ = 0.00004 for PC aa C40:3). Death from prostate cancer was associated with higher concentrations of acylcarnitine C3, amino acids methionine and *trans*-4-hydroxyproline, biogenic amine ADMA, hexose and sphingolipid SM (OH) C14:1 and lower concentration of glycerophospholipid PC aa C42:4.

**Conclusions:**

Several metabolites, i.e. C18:1, citrulline, *trans*-4-hydroxyproline, three glycerophospholipids and SM (OH) C14:1, might be related to prostate cancer. Analyses by time to diagnosis indicated that citrulline may be a marker of subclinical prostate cancer, while other metabolites might be related to aetiology. Several glycerophospholipids were inversely related to advanced stage disease. More prospective data are needed to confirm these associations.

**Electronic supplementary material:**

The online version of this article (doi:10.1186/s12916-017-0885-6) contains supplementary material, which is available to authorized users.

## Background

Prostate cancer is the second most commonly diagnosed cancer in men worldwide [1], but circulating insulin-like growth factor I is the only established risk factor that is potentially modifiable [2]. Examination of the metabolome may help us identify novel risk factors for prostate cancer [3, 4]. Metabolomics is the identification and quantification of metabolites (i.e. low molecular weight reactants, intermediates or products of biochemical reactions) in a biological system, and it is estimated that the human metabolome comprises many thousands of metabolites [5, 6]. Since metabolite concentrations are affected by dietary, lifestyle, environmental and genetic factors, the measurements provide a snapshot of biological activity [4, 7].

Little is known about how pre-diagnostic metabolite profiles relate to risk of prostate cancer [8–11]. A lower risk of overall and aggressive prostate cancer in men with higher serum concentrations of metabolites related to energy and lipid metabolism (including α-ketoglutarate, 1-stearoylglycerol and glycerophospholipids) has been reported [8, 9]. Similarly, a lower risk of overall prostate cancer has been suggested in men with higher plasma concentrations of some glycerophospholipids (lysophosphatidylcholines), while a positive association with risk has been indicated for the glycerophospholipid phosphatidylcholine (PC) acyl-alkyl (ae) C30:0, two amino acids and a biogenic amine [10]. Contrasting results have been found in a population screened for prostate cancer, including positive associations with lipids and inverse associations with amino acids and peptides [11]. The four published prospective studies, however, have a relatively limited number of cases (each less than 380). Only three of these studies have reported on prostate cancer risk by tumour characteristics [8, 9, 11], and in each, advanced stage and high grade tumours were considered together as one category of aggressive disease rather than separately [8, 9, 11]. As far as we are aware, there are no published prospective data on metabolite concentrations and subsequent risk of death from prostate cancer.

We report here the results from a large case-control study nested within the European Prospective Investigation into Cancer and Nutrition (EPIC) in which we aimed to prospectively investigate the association between metabolite concentrations and risk of prostate cancer, overall and by time to diagnosis and tumour characteristics, and risk of death from prostate cancer.

## Methods

### Study population

EPIC is a multi-centre cohort study comprising 520,000 men and women from ten European countries recruited between 1992 and 2000 [12]. It was designed to investigate how diet (intake and biomarkers) is associated with risk of cancer and other diseases. Among other important findings, it has helped establish insulin-like growth factor I as a risk factor for prostate cancer [2, 13].

The 153,400 men in the cohort were recruited from 19 centres in eight countries (Denmark, Germany, Greece, Italy, the Netherlands, Spain, Sweden and the UK). At recruitment, detailed information was collected on dietary intake, lifestyle, anthropometry and previous disease, and 139,600 men also gave a blood sample [12].

All participants gave written informed consent to participate in the EPIC cohort, and the EPIC study protocol was approved by the ethical committees of the International Agency for Research on Cancer (IARC), Lyon, France, and the participating centres. Approval for the current study was obtained from the Internal Review Board of the IARC (Project No. 14-09) and local ethics committees (see Declarations).

For the current analysis, men were eligible if they had blood stored at the central biobank at the IARC (centres in Germany, Greece, Italy, the Netherlands, Spain and the UK), the date of blood collection was known and if no cancer (except non-melanoma skin cancer) had been diagnosed at the time of blood collection.

#### Follow-up and case and control selection

Information on cancer incidence, tumour subtypes and vital status was obtained via record linkage to regional and national cancer registries, except in Germany and Greece where active follow-up was used and self-reported information was verified via health insurance or medical records and municipality-, hospital- and physician-based cancer and pathology registries and reports.

Prostate cancer was defined as code C61 in the 10^th^ revision of the International Statistical Classification of Diseases and Related Health Problems (ICD-10), and cases were men diagnosed with prostate cancer after blood collection and prior to the end of follow-up. For these analyses, samples were available for cases from both the first round of centralisation of follow-up data and a later round of follow-up (with end of follow-up ranging between centres from 2001 to 2002 and from 2007 to 2008, respectively).

Histological grade was known for 83.8% of cases, and 778 and 124 men were diagnosed with low-intermediate (Gleason score <8 or coded as well, moderately or poorly differentiated tumours) and high grade disease (Gleason score ≥8 or coded as undifferentiated tumours), respectively. Information on tumour stage was available for 61.7% of cases; 456 and 208 men had localised (tumour-node-metastasis (TNM) system score of ≤ T_2_ and N_0/x_ and M_0_, or stage coded as localised), and advanced stage tumours (TNM score of T_3–4_ and/or N_1–3_ and/or M_1_, or coded as advanced), respectively, and 115 men had aggressive prostate cancer (a subset of advanced stage disease defined as TNM score of T_4_ and/or N_1–3_ and/or M_1_), while 549 had non-aggressive disease.

Each case was matched to one control participant, selected randomly among male cohort participants who were alive and free of cancer (except non-melanoma skin cancer) at the time of diagnosis of the case. Matching criteria were study centre, length of follow-up, age (±6 months), time of day (±1 h) and fasting status (<3, 3–6, >6 h) at blood collection. An incidence density sampling procedure was used such that a control could become a case at a later date or be a control for more than one case.

### Blood collection and laboratory analysis

A standardised protocol for blood collection and processing was followed, and fasting was not required; details are published elsewhere [12]. All plasma samples (citrate anticoagulant) were assayed at the IARC, using the Absolute*IDQ* p180 Kit (Biocrates Life Sciences AG, Innsbruck, Austria) and following the procedure recommended by the vendor. A triple quadrupole mass spectrometer (Triple Quad 4500; AB Sciex, Framingham, MA, USA) was used to quantify a total of 142 metabolites. Samples from matched case-control sets were assayed in the same analytical batch, each of which included six to eleven quality control samples of pooled plasma. Laboratory personnel were blinded to sample category, i.e. case, study control or quality control.

The concentration of total prostate-specific antigen (PSA) at baseline was measured for a previous study [14] (Additional file 1) and was available for 71.1% of men in the current study, including 764 controls, for whom 489 had a concentration below 1 ng/ml, and 768 cases.

### Exclusion of participants and metabolites

Metabolite data were available for 2169 men (Additional file 2: Figure S1A). Metabolites were excluded if more than 15% of men had non-quantifiable assay results (missing data or results outside the measurable range; *n* = 18) or if the overall coefficient of variation (CV) was higher than 20% (*n* = 2; Additional file 1; Additional file 2: Figure S1B; Additional file 3: Table S1 shows the completeness of assay results and CVs). This left 122 metabolites for the analysis (7 acylcarnitines, 21 amino acids, 6 biogenic amines, 75 glycerophospholipids (all of which were phosphatidylcholines, the most abundant phospholipid in humans [15], denoted lysoPC or PC; for metabolite nomenclature see Additional file 1), hexose and 12 sphingolipids (all of which were sphingomyelins and denoted SM). Men with missing information on any of the 122 metabolites (*n* = 1) and men in incomplete case-control sets were excluded (*n* = 14), leaving 1077 matched case-control sets in the statistical analysis.

### Statistical analysis

Logarithmically transformed metabolite concentrations were used for all analyses.

Partial correlations were calculated between log-transformed concentrations of total PSA and metabolites separately in controls, controls with total PSA <1 ng/ml and cases, adjusting for age at blood collection (<55, 55–59, 60–64, 65–69, ≥70 years), body mass index (BMI; fourths, unknown) and study centre.

We used conditional logistic regression to estimate risk of prostate cancer per standard deviation (SD) increase in metabolite concentrations. Tests for linear trend were computed for metabolite concentrations as continuous variables. Departure from linearity was tested using the likelihood ratio χ^2^ test comparing models with the metabolite concentration as a linear term and as a cubic polynomial, respectively. The analysis was conditioned on the matching variables and further adjusted for exact age (continuously) in one model, and additionally for BMI (fourths, unknown), smoking (never, past, current, unknown), alcohol intake (<10, 10–19, 20–39, ≥40 g of alcohol per day, unknown), education (primary, secondary, degree level, unknown) and marital status (married or cohabiting, not married or cohabiting, unknown) in a second model. Results from the two models did not materially differ, and only results from the latter model are presented. A model based on fifths of metabolite concentrations was also computed (Additional file 1).

Similar conditional logistic regression models were fitted for subgroups by time to diagnosis (≤5 vs. >5 years) and tumour characteristics (low-intermediate vs. high grade, localised vs. advanced stage and non-aggressive vs. aggressive disease), and heterogeneity by subgroups was tested (Additional file 1).

In a sensitivity analysis, we excluded men who were also in a previous analysis conducted in EPIC-Heidelberg [10] (91 cases and 11 controls; personal communication, Tilman Kühn) and resulting in incomplete matched sets. This left 985 matched sets for this analysis.

All tests of statistical significance were two-sided, and to account for multiple testing the false discovery rate was controlled to 0.05 using the Benjamini-Hochberg method [16] (Additional file 1). All analyses were conducted in the Stata Statistical Software Package, version 14 (Stata Corporation, College Station, TX, USA).

## Results

At blood collection, participants were on average 60 years of age (range 40–77 years), and the men classified as cases were on average 67 years old at diagnosis (range 47–88 years). No clear differences were seen in baseline characteristics between cases and controls (Table 1).

**Table 1:** Characteristics of 1077 prostate cancer cases and 1077 controls

Characteristic	Cases, *n* = 1077	Controls, *n* = 1077
Age at blood collection, years (SD)	60.0 (7.1)	60.0 (7.1)
Height, cm (SD)^a^	171.7 (6.9)	172.1 (7.1)
BMI, kg/m^2^ (SD)^a^	26.8 (3.5)	26.9 (3.5)
Smoking, *n* (%)^a^		
Never	349 (33.0)	307 (29.0)
Former	467 (44.1)	501 (47.3)
Current	242 (22.9)	252 (23.8)
Alcohol consumption, *n* (%)^a^		
< 10 g/day	479 (44.8)	480 (44.6)
10–19 g/day	199 (18.6)	201 (18.7)
20–40 g/day	218 (20.4)	221 (20.5)
≥ 40 g/day	174 (16.3)	174 (16.2)
Physical activity, *n* (%)^a^		
Inactive	292 (28.0)	273 (26.0)
Moderately inactive	328 (31.4)	326 (31.1)
Moderately active	242 (23.2)	246 (23.5)
Active	182 (17.4)	204 (19.4)
Marital status, *n* (%)^a^		
Married or cohabiting	718 (89.4)	730 (90.2)
Not married or cohabiting	85 (10.6)	79 (9.8)
Educational attainment, *n* (%)^a^		
Primary or equivalent	413 (41.1)	425 (42.2)
Secondary	369 (36.7)	396 (39.4)
Degree	223 (22.2)	185 (18.4)
Cases only		
Age at diagnosis, years (SD)	66.9 (7.0)	–
Time to diagnosis, *n* (%)^b^		
< 2 years	137 (12.7)	–
2 to <4 years	177 (16.4)	–
4 to <6 years	191 (17.7)	–
6 to <8 years	120 (11.1)	–
8 to <10 years	136 (12.6)	–
≥ 10 years	316 (29.3)	–
Year of diagnosis, median (range)	2001 (1994-2008)	–
Grade, *n* (%)^a,c^		
Low-intermediate grade	778 (86.3)	–
High grade	124 (13.7)	–
Stage, *n* (%)^a,d^		
Localised	456 (68.7)	–
Advanced	208 (31.3)	–
Non-aggressive	549 (82.7)	–
Aggressive	115 (17.3)	–
Death from prostate cancer, *n* (%)^a,e^	127 (12.3)	–

^a^Unknown values for some participants; the calculations of percentages exclude missing values

^b^Time between blood collection and diagnosis

^c^Gleason score <8 or coded as well, moderately or poorly differentiated for low-intermediate grade and Gleason score ≥8 or coded as undifferentiated for high grade

^d^The TNM system was used to categorise stages of prostate cancer; localised: ≤T_2_ and N_0/x_ and M_0_, or coded as localised; advanced: T_3–4_ and/or N_1–3_ and/or M_1_, or coded as advanced; and aggressive: T_4_ and/or N_1–3_ and/or M_1_. All categories are not mutually exclusive, so numbers do not add up; percentages were calculated separately for localised and advanced, and for non-aggressive and aggressive

^e^Death from prostate cancer (prostate cancer listed as the underlying cause of death on the death certificate) during follow-up; 144 died of prostate cancer, but 17 were excluded from further analysis as their matched control had died before them (*n* = 13) or vital status was not known for their control (*n* = 4)

The distribution of metabolite concentrations by case-control status is shown in Additional file 3: Table S2.

Strong positive correlations were observed within metabolite classes and between glycerophospholipids and sphingolipids (Additional file 2: Figure S2). Total PSA and metabolite concentrations were not strongly correlated in controls, controls with low total PSA concentration or in cases (–0.16 ≤ *r* ≤ 0.13; Additional file 2: Figure S2; Additional file 3: Table S3).

### Overall prostate cancer

**Fig. 1:**
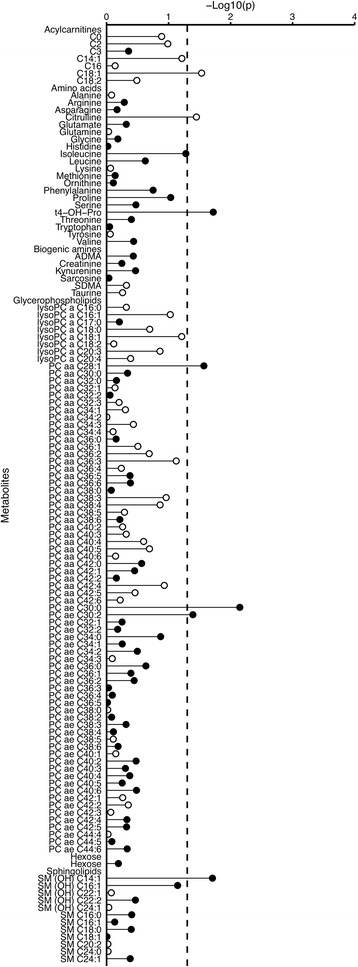
Statistical significance of associations between metabolite concentrations and risk of overall prostate cancer. The analysis included 1077 matched case-control sets. Statistical significance was plotted as –log_10_(*p* values). The *dashed line* represents conventionally statistical significance at α = 0.05. *Filled circles* represent positive associations, and *unfilled circles* represent inverse associations. The *p* values were derived from a conditional logistic regression using log metabolite concentration as a continuous variable and adjusting for exact age (continuously), body mass index (fourths; unknown), smoking (never; past; current; unknown), alcohol intake (<10; 10–19; 20–39; ≥40 g of alcohol per day; unknown), education (primary or none; secondary; degree level; unknown) and marital status (married or cohabiting; not married or cohabiting; unknown)

**Fig. 2:**
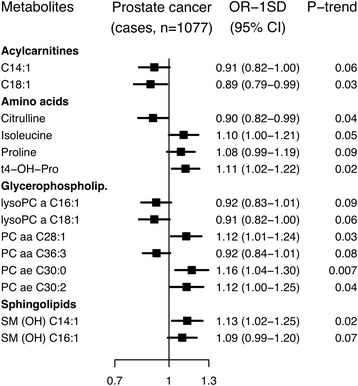
Odds ratios for overall prostate cancer risk by concentration of selected metabolites. Metabolites with *p* for linear trend <0.1 were included in the figure; no associations were statistically significant after controlling the false discovery rate at α = 0.05 (Benjamini-Hochberg). Odds ratios for one standard deviation increase in metabolite concentrations, 95% confidence intervals and *p* values for linear trend were derived from a conditional logistic regression using log metabolite concentration divided by the standard deviation of log metabolite concentration as a continuous variable and adjusting for exact age (continuously), body mass index (fourths; unknown), smoking (never; past; current; unknown), alcohol intake (<10; 10–19; 20–39; ≥40 g of alcohol per day; unknown), education (primary or none; secondary; degree level; unknown) and marital status (married or cohabiting; not married or cohabiting; unknown)

The statistical significance of associations between metabolite concentrations and overall prostate cancer risk is shown in Fig. 1, and in Fig. 2 odds ratios (ORs) and 95% confidence intervals (CIs) are shown for metabolites with *p* < 0.1 (additional results in Additional file 3: Tables S4 and S5). Conventionally statistically significant (*p* < 0.05) lower risks were seen in men with higher concentrations of the acylcarnitine C18:1 and the amino acid citrulline, while higher risks were seen for the amino acid *trans*-4-hydroxyproline (t4-OH-Pro), glycerophospholipids PC diacyl (aa) C28:1, PC ae C30:0 and PC ae C30:2 and sphingolipid SM (OH) C14:1, but none of the associations were statistically significant after controlling for multiple testing. The strongest association was with PC ae C30:0, for which the risk was 16% higher per SD increase in concentration (OR_1SD_ = 1.16; 95% CI 1.04–1.30). There was no evidence of departure from linearity in the association with prostate cancer risk for these metabolites, except for C18:1 for which the test for non-linearity was conventionally significant, but not significant after controlling for multiple testing (Additional file 3: Table S4).

### Time to diagnosis

**Fig. 3:**
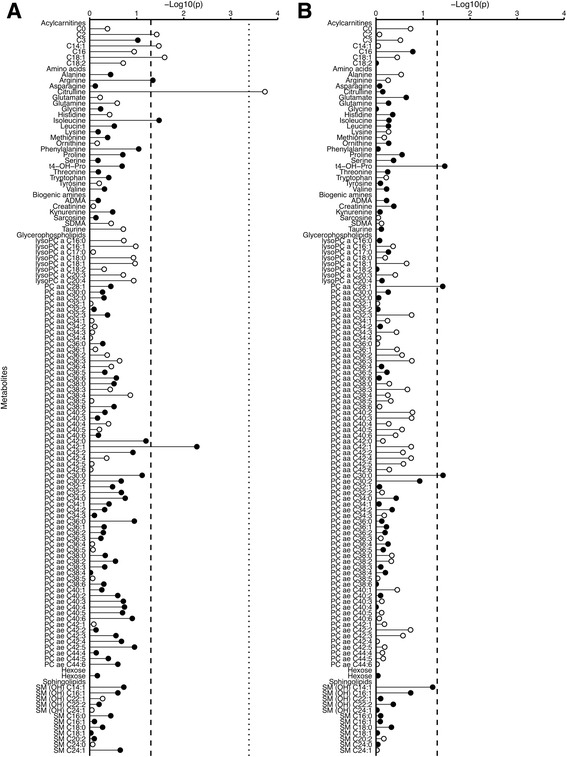
Statistical significance of associations between metabolite concentrations and prostate cancer risk by time to diagnosis. **a** Five years or less between blood collection and diagnosis; *n* = 428 matched case-control sets. **b** More than 5 years between blood collection and diagnosis; *n* = 649 matched sets. Statistical significance was plotted as –log_10_(*p* values). The *dashed* and the *dotted lines* represent conventionally statistical significance and statistical significance after controlling the false discovery rate (Benjamini-Hochberg), respectively, both at α = 0.05. *Filled circles* represent positive associations, and *unfilled circles* represent inverse associations. The *p* values were derived from a conditional logistic regression using log metabolite concentration as a continuous variable and adjusting for exact age (continuously), body mass index (fourths; unknown), smoking (never; past; current; unknown), alcohol intake (<10; 10–19; 20–39; ≥40 g of alcohol per day; unknown), education (primary or none; secondary; degree level; unknown) and marital status (married or cohabiting; not married or cohabiting; unknown)

Of the cases, 428 (39.7%) were diagnosed within 5 years of blood collection. A one SD increase in citrulline concentration was associated with a 27% lower risk of prostate cancer diagnosed within 5 years of blood collection but not with prostate cancer diagnosed later after blood collection (OR_1SD_ = 0.73; 95% CI 0.62–0.86; *p*_trend_ = 0.0002, which was significant after controlling for multiple testing, and OR_1SD_ = 1.02; 95% CI 0.90–1.16, respectively; *p*_heterogeneity_ = 0.0009; Fig. 3; Additional file 3: Table S6).

Conventionally significant associations were seen in the first 5 years of follow-up, inversely for three acylcarnitines (including C18:1) and positively for two amino acids and a glycerophospholipid, and for follow-up beyond 5 years, for t4-OH-Pro, PC aa C28:1 and PC ae C30:0, although there was no evidence of heterogeneity by time to diagnosis.

### High grade

Conventionally significant inverse associations with high grade prostate cancer were seen for an acylcarnitine, 24 glycerophospholipids and three sphingolipids, with risk reductions up to 48% (OR_1SD_ = 0.52, 95% CI 0.35–0.79 for PC aa C32:3; Fig. 4; Additional file 2: Figure S3; Additional file 3: Table S7); however, the associations did not remain after correcting for multiple testing.

**Fig. 4:**
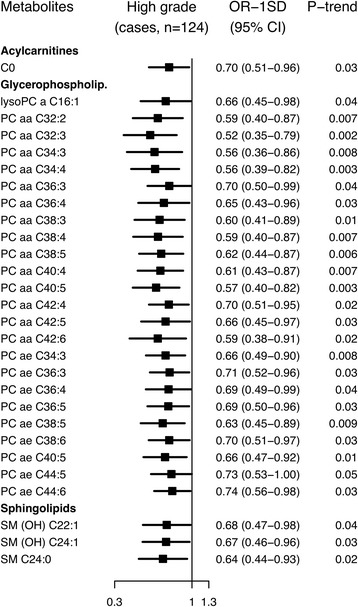
Odds ratios for high grade prostate cancer by concentration of selected metabolites. Tumours with Gleason score ≥8 or coded as undifferentiated were defined as high grade. Metabolites with *p* for linear trend <0.05 were included in the figure; no associations were statistically significant after controlling the false discovery rate at α = 0.05 (Benjamini-Hochberg). Odds ratios for one standard deviation increase in metabolite concentrations, 95% confidence intervals and *p* values for linear trend were derived from a conditional logistic regression using log metabolite concentration divided by the standard deviation of log metabolite concentration as a continuous variable and adjusting for exact age (continuously), body mass index (fourths; unknown), smoking (never; past; current; unknown), alcohol intake (<10; 10–19; 20–39; ≥40 g of alcohol per day; unknown), education (primary or none; secondary; degree level; unknown) and marital status (married or cohabiting; not married or cohabiting; unknown)

### Advanced stage

After controlling for multiple testing, 12 glycerophospholipids, i.e. lysoPC a C18:0, PC aa C36:2, PC aa C36:3, PC aa C38:3, PC aa C38:5, PC aa C40:2, PC aa C40:3, PC aa C40:4, PC aa C40:5, PC aa C42:4, PC aa C42:5 and PC ae C40:1, were inversely associated with risk of advanced prostate cancer, with risk reductions up to 46% (OR_1SD_ = 0.54; 95% CI 0.40–0.72 for PC aa C40:3; Fig. 5; Additional file 2: Figure S4; Additional file 3: Table S8). For six of these and three additional glycerophospholipids, the test for heterogeneity by stage was significant after taking multiple testing into account, with inverse associations for advanced disease and no associations for localised disease.

**Fig. 5:**
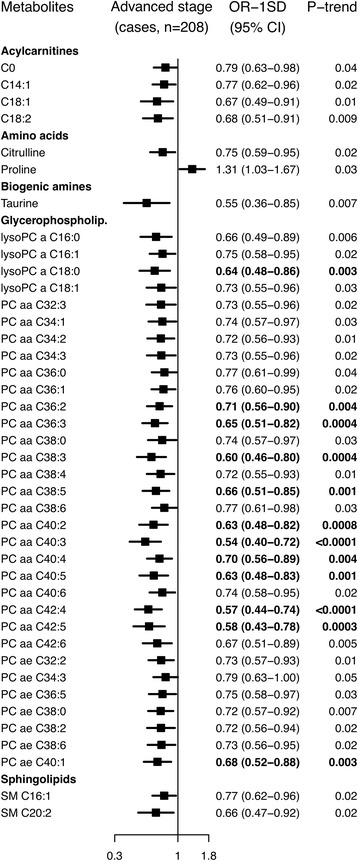
Odds ratios for advanced stage prostate cancer by concentration of selected metabolites. Advanced stage tumours were defined as T_3–4_ and/or N_1–3_ and/or M_1_, using the tumour-node-metastasis staging system. Metabolites with *p* for linear trend <0.05 were included in the figure, and values marked in *boldface* were statistically significant after allowing for multiple testing using a false discovery rate controlling procedure at α = 0.05 (Benjamini-Hochberg). Odds ratios for one standard deviation increase in metabolite concentrations, 95% confidence intervals and *p* values for linear trend were derived from a conditional logistic regression using log metabolite concentration divided by the standard deviation of log metabolite concentration as a continuous variable and adjusting for exact age (continuously), body mass index (fourths; unknown), smoking (never; past; current; unknown), alcohol intake (<10; 10–19; 20–39; ≥40 g of alcohol per day; unknown), education (primary or none; secondary; degree level; unknown) and marital status (married or cohabiting; not married or cohabiting; unknown)

Additionally, conventionally significant associations with advanced disease were seen for four acylcarnitines, two amino acids, a biogenic amine, 20 glycerophospholipids and two sphingolipids; all but the amino acid proline were inversely associated with advanced disease.

### Aggressive prostate cancer

**Fig. 6:**
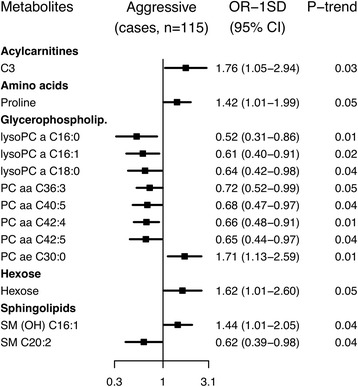
Odds ratios for aggressive prostate cancer by concentration of selected metabolites. Aggressive tumours were defined as T_4_ and/or N_1–3_ and/or M_1_, using the tumour-node-metastasis staging system. Metabolites with *p* for linear trend <0.05 were included in the figure; no associations were statistically significant after controlling the false discovery rate at α = 0.05 (Benjamini-Hochberg). Odds ratios for one standard deviation increase in metabolite concentrations, 95% confidence intervals and *p* values for linear trend were derived from a conditional logistic regression using log metabolite concentration divided by the standard deviation of log metabolite concentration as a continuous variable and adjusting for exact age (continuously), body mass index (fourths; unknown), smoking (never; past; current; unknown), alcohol intake (<10; 10–19; 20–39; ≥40 g of alcohol per day; unknown), education (primary or none; secondary; degree level; unknown) and marital status (married or cohabiting; not married or cohabiting; unknown)

Conventionally significant associations were seen for 13 metabolites with aggressive prostate cancer. Positive associations were observed with an acylcarnitine, an amino acid, a glycerophospholipid, hexose and a sphingomyelin, while inverse associations were seen for seven glycerophospholipids and a sphingolipid, but none remained after controlling the false discovery rate (Fig. 6; Additional file 2: Figure S5; Additional file 3: Table S9). The strongest association was with glycerophospholipid lysoPC a C16:0, for which the estimated risk reduction was 48% (OR_1SD_ = 0.52, 95% CI 0.31–0.86).

### Death from prostate cancer

During follow-up, 144 men died of prostate cancer, and after excluding matched sets in which the control had died before the case (*n* = 13) or vital status was unknown for the control (*n* = 4), 127 matched sets were available for analysis. Seven metabolites were conventionally significantly associated with death from prostate cancer. Men with higher concentrations of an acylcarnitine (C3), two amino acids (methionine and t4-OH-Pro), a biogenic amine (ADMA), hexose and a sphingolipid (SM (OH) C14:1) were at higher risk, while an inverse association was found for a glycerophospholipid (PC aa C42:4; Fig. 7; Additional file 2: Figure S6; Additional file 3: Table S10). However, the associations did not remain after controlling for multiple testing.

**Fig. 7:**
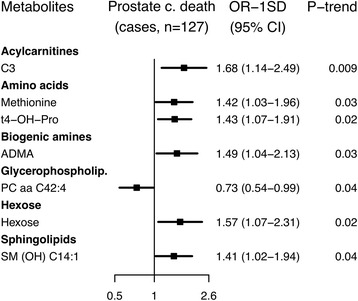
Odds ratios for death from prostate cancer by selected concentration of metabolites. Metabolites with *p* for linear trend <0.05 were included in the figure; no associations were statistically significant after controlling the false discovery rate at α = 0.05 (Benjamini-Hochberg). Odds ratios for one standard deviation increase in metabolite concentrations, 95% confidence intervals and *p* values for linear trend were derived from a conditional logistic regression using log metabolite concentration divided by the standard deviation of log metabolite concentration as a continuous variable and adjusting for exact age (continuously), body mass index (fourths; unknown), smoking (never; past; current; unknown), alcohol intake (<10; 10–19; 20–39; ≥40 g of alcohol per day; unknown), education (primary or none; secondary; degree level; unknown) and marital status (married or cohabiting; not married or cohabiting; unknown

### Sensitivity analysis

Results were not materially changed after excluding the overlap in participants between the EPIC-Heidelberg study [10] and our analysis (Additional file 3: Table S11).

## Discussion

### Main findings

In this prospective study of 122 plasma metabolite concentrations and prostate cancer risk, seven metabolites were associated with risk of overall prostate cancer at a conventional level of significance but not after correction for multiple testing; these were from several metabolite classes, suggesting that dysregulation of many metabolic pathways may be related to prostate cancer. The results stratified by time to diagnosis suggested that lower concentrations of citrulline might be a marker of subclinical prostate cancer, as the association was observed for disease diagnosed in the first few years after blood draw only. In contrast, the associations with others, including t4-OH-Pro, SM (OH) C14:1, PC ae C30:0 and other glycerophospholipids, did not vary by time to diagnosis, and these might thus provide insights into aetiology. Subgroup analysis indicated a possible link between higher concentrations of several glycerophospholipids and decreased risk of high risk tumour subtypes, especially advanced stage prostate cancer. Similar to the results for overall prostate cancer, suggested associations were observed between death from prostate cancer and seven metabolites from several metabolite classes.

### Other studies

Besides the current study, four smaller prospective studies of metabolomics and prostate cancer risk have been published; two were nested within the Alpha-Tocopherol, Beta-Carotene Cancer Prevention Study (ATBC) [8, 9], one in the EPIC-Heidelberg study [10] and one in the Prostate, Lung, Colorectal, and Ovarian Cancer Screening Trial (PLCO) [11] (with 74, 200, 310 and 380 cases, respectively). Comparison of results from metabolomics studies is not straightforward owing to differences in population characteristics, biological medium [17, 18], the technological assay and statistical tools, including procedures for dealing with the large number of metabolites. Nonetheless, replication of possible associations both with specific metabolites and also more globally with metabolite classes is essential for interpretation of results.

In the two ATBC analyses, mass spectrometry was used to measure 420 and 626 metabolites, respectively, samples were fasting serum samples from smokers and stage and grade were combined to define aggressive tumours (as opposed to our more restricted definition of aggressive disease based on tumour stage, nodes and metastasis but not grade) [8, 9]. Among other metabolite classes, amino acids, glycerophospholipids and sphingolipids were measured and with some overlap of specific metabolites with the current study (19 amino acids and two biogenic amines). The first ATBC analysis [8] did not find a significant association between citrulline and prostate cancer, but for overall prostate cancer the OR per SD increase (0.87, 95% CI 0.62–1.23) was similar to the observation in our study (0.90, 95% CI 0.82–0.99). We did not replicate the ATBC results indicative of a lower risk of prostate cancer (*p* < 0.05) in men with higher concentrations of some amino acids, i.e. alanine (for overall and aggressive prostate cancer), lysine (for overall and non-aggressive prostate cancer), methionine (for overall and aggressive prostate cancer) or phenylalanine (for overall and non-aggressive prostate cancer). In contrast, we observed a conventionally significant higher risk of death from prostate cancer with higher methionine concentration. The main finding of the first ATBC study was a strong inverse association between 1-stearoylglycerol, a product of lipid breakdown, and risk of overall and aggressive prostate cancer, but we did not have data on this metabolite. In line with the first ATBC analysis, the second analysis [9], with no overlap in participants, showed inverse associations of lipids (including glycerophospholipids) and energy metabolites (involved in the Krebs cycle) with risk of aggressive prostate cancer. Similarly, we found inverse associations for several glycerophospholipids with risk for high grade, aggressive (defined based on stage) and especially advanced stage prostate cancer.

The PLCO analysis used the same metabolomic assay as the ATBC analyses, although on non-fasting rather than fasting serum samples, but in contrast to any of the other studies, participants were all screened for prostate cancer using an annual PSA test and digital rectal examination [11]. A lower risk of prostate cancer was suggested (*p* < 0.05) in relation to higher concentrations of some amino acids and their derivatives, including arginine (with aggressive prostate cancer; defined by combining stage and grade information) and tryptophan (with overall and aggressive prostate cancer). We did not replicate these findings. The reported positive associations with lipids for overall and aggressive prostate cancer were inconsistent with the results from ATBC and our findings. These differences may be due to the screening of the PLCO population [11], as screening was not common in ATBC [9] or EPIC.

The EPIC-Heidelberg analysis [10] mostly investigated the same metabolites as we did and used a similar assay. The strongest finding for overall prostate cancer in both studies was a suggested (*p* < 0.05) positive association with the glycerophospholipid PC ae C30:0 (EPIC-Heidelberg: OR_top vs. bottom fourth_ = 1.89, 95% CI 1.06–3.36). Our results were not materially changed after excluding the small overlap in participants between the two analyses. In both EPIC-Heidelberg and our analyses, the results for PC ae C30:0 did not differ by time to diagnosis. Additionally, the EPIC-Heidelberg analysis suggested inverse associations with three glycerophospholipids (all lysophosphatidylcholines) and positive associations with alanine, proline and methionine sulphoxide, the latter of which was not measured in our analysis. Our results for these metabolites were mainly in the same direction but less strong for overall prostate cancer, while we observed stronger inverse associations for two of the lysophosphatidylcholines with aggressive disease.

### Possible mechanisms

The strongest findings in our study were an inverse association of citrulline with risk of prostate cancer diagnosis within the first 5 years of follow-up, suggested positive associations of prostate cancer risk with t4-OH-Pro, SM (OH) C14:1 (for overall and death from prostate cancer for both metabolites) and PC ae C30:0 (for overall and aggressive prostate cancer), and the lower risk of advanced prostate cancer in relation to glycerophospholipid concentrations. However, relatively little is known about the potential biological role of these specific metabolites in carcinogenesis of the prostate.

Citrulline has antioxidant functions and has been shown to protect DNA and enzymes from reactive oxygen species [19], which might otherwise promote progression of prostate cancer via continuous proliferation and impaired apoptosis [20]. In line with our results, lower urinary concentrations of citrulline have been observed in patients with prostate cancer compared to healthy controls [21], perhaps because of altered citrulline metabolism in tumour cells. However, whether citrulline might be useful as a marker of subclinical prostate cancer needs to be confirmed.

It is not clear why t4-OH-Pro (one variant of hydroxyproline) might be related to risk of prostate cancer, but urinary excretion of hydroxyproline has been previously recognised as an early marker of bone metastases in patients with prostate cancer [22, 23], as hydroxyproline is released from collagen in tumour invasion [24]. While 4-hydroxyproline has also been suggested as a marker of red and processed meat [25, 26], the evidence does not suggest an association between these foods and prostate cancer risk [27].

Standard amino acids have often been reported to differ between controls and patients with various cancer types [4, 28], but it is not clear if the associations with the non-standard amino acids citrulline and t4-OH-Pro are specific to prostate cancer or not.

A role of sphingomyelins (the type of sphingolipid investigated here) in carcinogenesis might be explained by their involvement in cell proliferation, migration and autophagy [29]. Higher sphingomyelin concentrations in prostate tumour tissue and patients’ plasma than in benign prostatic hyperplasia tissue and control participants, respectively, have been reported [30, 31]. SM (OH) C14:1 has also been suggested as a marker of cream intake [25], and intake of dairy products might be related to prostate cancer risk [32].

All glycerophospholipids investigated here were phosphatidylcholines, the homeostasis of which (including PC ae C30:0) plays a critical role in cell regulation, with increased synthesis leading to proliferation [33]. Higher plasma phosphatidylcholine concentrations in patients with prostate cancer than in controls have been reported [31], which is in line with the positive association of PC ae C30:0 with risk. While possible mechanisms for the inverse associations between several phosphatidylcholines and advanced prostate cancer are not clear, positive associations have been reported between concentrations of diacyl-phosphatidylcholines (denoted PC aa Cx:y in the current paper) and type 2 diabetes [34], which is linked to a lower prostate cancer risk [35, 36]. The association between phosphatidylcholines and risk might apply to malignancies in general rather than being prostate cancer specific. Changes in phosphatidylcholine concentrations have been reported in patients with cancers of the bladder, brain, breast, kidney, liver, lung and ovaries [4, 28, 37], and in the prospective analysis in the EPIC-Heidelberg subcohort, associations with breast and colorectal cancer were also suggested [10]. Altered circulating phosphatidylcholine concentrations in men who are subsequently diagnosed with cancer may be due to altered lipid uptake and metabolism by rapidly proliferating cancer cells in subclinical tumours; these metabolites are required for membrane synthesis and lipid-based cell signalling [38].

### Strengths and limitations

To date, this is the largest study of metabolite concentrations and risk of prostate cancer. The relatively large number of participants has enabled us to investigate not only total risk of prostate cancer but also to conduct exploratory analyses by time to diagnosis and prostate tumour characteristics, and of death from prostate cancer. Furthermore, the detailed information on covariates has reduced the risk of confounding driving the results.

The choice of metabolomics assay was determined by coverage of metabolites of a priori interest (amino acids) and cost, but use of a targeted assay limits our analysis to the metabolites covered by the assay.

The study has a number of other limitations. Only one blood sample was available per participant, which will attenuate the results if a single measure does not represent long-term exposure. Reproducibility of metabolites over 4 months to 2.3 years has been reported to be moderate to high (median interclass correlation coefficients: 0.54–0.70), suggesting that a single measurement may be adequate for most metabolites [18, 39, 40], although the reproducibility was lower for some acylcarnitines and a few amino acids [18, 39]. Secondly, pre-analytical conditions, e.g. food consumption prior to blood collection and use of anticoagulant in blood sampling tubes, could potentially diminish our ability to detect associations [41–43]. However, fasting status has been found to explain only a small amount of the variability of metabolite concentrations [41, 44–46], and cases and controls were matched on time since last food or drink to minimise any risk of bias due to fasting status. Similarly, cases and controls were matched by centre as a proxy for sample handling. Like other anticoagulants, use of citrate in plasma samples can affect measurements of metabolites, including amino acids, glycerophospholipids and sphingomyelins [42, 43]. Thus, the metabolite concentrations reported here might not be directly comparable to concentrations measured in serum, or in plasma treated with EDTA or heparin. However, relative risk estimates are unlikely to be affected, as citrate was used in all samples. Thirdly, variation in the histological grading and stage classification between pathologists and over time could result in some misclassification of tumours by stage and grade category [47–50], which in turn might lead to attenuation of risk estimates in subgroup analyses. Finally, although this is the largest study to date of metabolites and prostate cancer risk, the numbers and thus the statistical power are still limited, especially for analyses of tumour subtypes and death. Furthermore, a relatively conservative controlling procedure for multiple testing which does not account for correlations between metabolites was used.

### Future research

The inclusion of data from further incident prostate cancer cases and matched controls in future analyses will increase the reliability of estimates of the associations between metabolite concentrations and prostate cancer risk, overall and for tumour subtypes. Dimension-reduction approaches that allow the investigation of patterns in metabolite profile may also provide further insights into the role of plasma metabolites in prostate cancer development.

## Conclusions

This large study of pre-diagnostic plasma metabolites and prostate cancer risk suggested that several metabolites, including acylcarnitines, amino acids, glycerophospholipids and sphingolipids, might be related to prostate cancer. Analyses stratifying for time to diagnosis indicated that low concentrations of citrulline might be a marker of subclinical prostate cancer, while other metabolites might be related to aetiology. Higher concentrations of several glycerophospholipids might be associated with lower risk of advanced stage prostate cancer. These results need to be further investigated in other large prospective studies with data on prostate tumour characteristics and death.

## Additional files

Additional file 1: Supplementary methods, including additional information on laboratory and statistical analyses. (PDF 84 kb)

Additional file 2: Supplementary figures, including flow chart of exclusions (**Figure S1A** and **B**); correlations between metabolite and total PSA concentrations (**Figure S2**); and statistical significance of associations between metabolite concentrations and high grade prostate cancer (**Figure S3**), advanced stage prostate cancer (**Figure S4**), aggressive prostate cancer (**Figure S5**) and death from prostate cancer (**Figure S6**). (PDF 805 kb)

Additional file 3: Supplementary results on completeness and analytical quality of the metabolomics assay (**Table S1**); distributions of metabolite concentrations by case-control status (**Table S2**); correlations between total PSA and metabolite concentrations (**Table S3**); overall risk of prostate cancer per one standard deviation increase in metabolite concentration (**Table S4**) and by fifths of metabolite concentration (**Table S5**); risk of prostate cancer by time to diagnosis (**Table S6**); risk of prostate cancer by tumour characteristics, i.e. risk by low-intermediate and high grade (**Table S7**), localised and advanced stage (**Table S8**) and non-aggressive and aggressive disease (**Table S9**); risk of death from prostate cancer (**Table S10**); and risk of overall prostate cancer after excluding participants who were also in an analysis within EPIC-Heidelberg (**Table S11**). (XLSX 203 kb)

## Abbreviations

aa: Diacyl
ae: Acyl-alkyl
ATBC: Alpha-Tocopherol, Beta-Carotene Cancer Prevention Study
BMI: Body mass index﻿
﻿CI: Confidence interval
CV: Coefficient of variation
Cx:y: x carbon atoms and y double bonds in fatty acids
EPIC: European Prospective Investigation into Cancer and Nutrition
IARC: International Agency for Research on Cancer
LOD: Limit of detection
LOQ: Limit of quantification
OH: Hydroxy
OR: Odds ratio
PC: Phosphatidylcholine
PLCO: Prostate, Lung, Colorectal, and Ovarian Cancer Screening Trial
PSA: Prostate-specific antigen
SD: Standard deviation
SM: Sphingomyelin
t4-OH-pro: *Trans*-4-hydroxyproline
TNM: Tumour-node-metastasis
