# Supplementary material for: Pre-diagnostic metabolite concentrations and prostate cancer risk in 1077 cases and 1077 matched controls in the European Prospective Investigation into Cancer and Nutrition
Source: BMC Med. 2017 Jul 5;15:122. doi: 10.1186/s12916-017-0885-6 (PMC5497352; doi:10.1186/s12916-017-0885-6)
Supplement: Supplementary file 1 — Supplementary methods, including additional information on laboratory and statistical analyses. (PDF 84 kb) [file 12916_2017_885_MOESM1_ESM.pdf]

ADDITIONAL FILE 1

SUPPLEMENTARY METHODS

Laboratory analysis

Assay of total prostate specific antigen (PSA)

Immunoassay measurements for total PSA were conducted using the dual-label DELFIA Prostatus® total PSA-Assay (Perkin-Elmer, Turku, Finland) [1] calibrated against the WHO 96/670 (PSA-WHO) standard in Dr. Lilja's laboratory at the Wallenberg Research Laboratories, Department of Translational Medicine, Lund University, Skåne University Hospital, Malmö, Sweden, for a previous study [2]. Intra- and inter-assay coefficients of variation were less than 9%. PSA concentration was available for 71.1% of men in the current study, including 764 controls, 489 of which had a concentration below 1 ng/ml, and for 768 cases.

Metabolite measurements outside the measurable range

After excluding 18 metabolites for which more than 15% of participants had measurements outside the measurable range (Additional file 3: Table S1), the remaining measurements outside the measurable range were imputed. Measurements below the limit of detection (applicable to 12 metabolites for 1 to 307 men) and quantification (applicable to 7 metabolites for 1 to 296 men) were set to half the lowest measured concentration and to half the limit of quantification, respectively. Measurements above the highest concentration calibration standards were set to the highest standard concentration (applicable to 1 metabolite for 1 man).

Coefficients of variation for metabolite concentrations

Overall coefficients of variations were calculated as the standard deviation divided by the mean (Additional file 3: Table S1). For the 122 included metabolites, the median (range) was 12.3% (7.0-17.2) for acylcarnitines, 8.8% (6.0-12.6) for amino acids, 10.6% (4.3-17.2) for biogenic amines, 11.2% (7.3-

17.7) for glycerophospholipids and 10.4% (8.0-19.9) for sphingolipids, and the coefficient of variation for hexose was 6.5%.

Nomenclature of metabolites

Fatty acid side chains in acylcarnitines, glycerophospholipids and sphingolipids were labelled “Cx:y,” where x and y denote the total number of carbon atoms and double bonds, respectively, in each molecule [3]. Acylcarnitines were abbreviated according to the fatty acid side chain. All glycerophospholipids were phosphatidylcholines, and subclasses were separated by the number and type of fatty acids side chains. “LysoPC a” denotes phosphatidylcholines with one acyl fatty acid side chain, “PC aa” denotes two acyl side chains, and “PC ae” denotes one acyl and one alkyl side chain. Sphingolipids were all sphingomyelins with a hydroxyl group (SM (OH)) or without a hydroxy group attached and were also labelled according to the fatty acid side chain. Hexose is the sum of a range of monosaccharides with six carbon atoms, including glucose, fructose and galactose.

Statistical analysis

Conditional logistic regression by fifths of metabolite concentrations

Conditional logistic regression was used to estimate risk of prostate cancer by fifths of metabolite concentrations (based on the distribution among controls). Tests for linear trend were computed across the median concentrations in the fifths. Like in the main model presented in the paper, this model was conditioned on the matching variables and further adjusted for exact age (continuously), body mass index (fourths; unknown), smoking (never; past; current; unknown), alcohol intake (<10; 10-19; 20-39; ≥40 g of alcohol per day; unknown), education (primary; secondary; degree level; unknown) and marital status (married or cohabiting; not married or cohabiting; unknown).

Test for heterogeneity

Tests for heterogeneity in the associations between metabolite concentrations and prostate cancer risk by subgroups (i.e. time to diagnosis and tumour characteristics) were done using the likelihood ratio χ^2 test, which compared models with and without an interaction term between the linear trend variables and the outcome variable of interest.

Multiple testing

The Benjamini-Hochberg false discovery rate controlling procedure was used to account for multiple testing in all analyses of metabolite concentrations.

First, the p-values were sorted and ranked from the lowest p(1) to the highest p(m). Let k be the largest rank (i) for which $p(i) < (i/m) \times \alpha$ is true; α is the significance level and set to 0.05 in all analyses. Then all the null hypotheses for p-values from p(1) to p(k) were rejected [4]. This method allows 5% of positive findings to be false, on average [5].

Additionally, an adjusted p-value (p_{adj} sometimes referred to as a q-value by other authors) was computed. $p_{adj}(i)$ was defined as the minimum of p(n) x (m/n) for n being i, i+1, …, m. tests with a $p_{adj} < 0.05$ were declared significant after controlling the FDR at 5% [6].

References

1. Mitrunen K, Pettersson K, Piironen T, Bjork T, Lilja H, Lovgren T. Dual-label one-step immunoassay for simultaneous measurement of free and total prostate-specific antigen concentrations and ratios in serum. Clin Chem. 1995;41:1115-20.

2. Byrne KS, Castaño JM, Chirlaque MD, Lilja H, Agudo A, Ardanaz E et al. Vasectomy and Prostate Cancer Risk in the European Prospective Investigation Into Cancer and Nutrition (EPIC). Journal of Clinical Oncology. 2017;35:1297-303.

3. Floegel A, Drogan D, Wang-Sattler R, Prehn C, Illig T, Adamski J et al. Reliability of serum metabolite concentrations over a 4-month period using a targeted metabolomic approach. PLoS One. 2011;6:e21103.

4. Benjamini Y, Hochberg Y. Controlling the False Discovery Rate: A Practical and Powerful Approach to Multiple Testing. Journal of the Royal Statistical Society Series B (Methodological). 1995;57:289-300.

5. Chadeau-Hyam M, Campanella G, Jombart T, Bottolo L, Portengen L, Vineis P et al. Deciphering the complex: methodological overview of statistical models to derive OMICS-based biomarkers. Environ Mol Mutagen. 2013;54:542-57.

6. Noble WS. How does multiple testing correction work? Nat Biotechnol. 2009;27:1135-7.
