# Supplementary material for: Pre-diagnostic metabolite concentrations and prostate cancer risk in 1077 cases and 1077 matched controls in the European Prospective Investigation into Cancer and Nutrition
Source: BMC Med. 2017 Jul 5;15:122. doi: 10.1186/s12916-017-0885-6 (PMC5497352; doi:10.1186/s12916-017-0885-6)
Supplement: Supplementary file 2 — Supplementary figures, including flow chart of exclusions (Figure S1A and B); correlations between metabolite and total PSA concentrations (Figure S2); and statistical significance of associations between metabolite concentrations and high grade prostate cancer (Figure S3), advanced stage prostate cancer (Figure S4), aggressive prostate cancer (Figure S5) and death from prostate cancer (Figure S6). (PDF 805 kb) [file 12916_2017_885_MOESM2_ESM.pdf]

# ADDITIONAL FILE 2

## SUPPLEMENTARY FIGURES

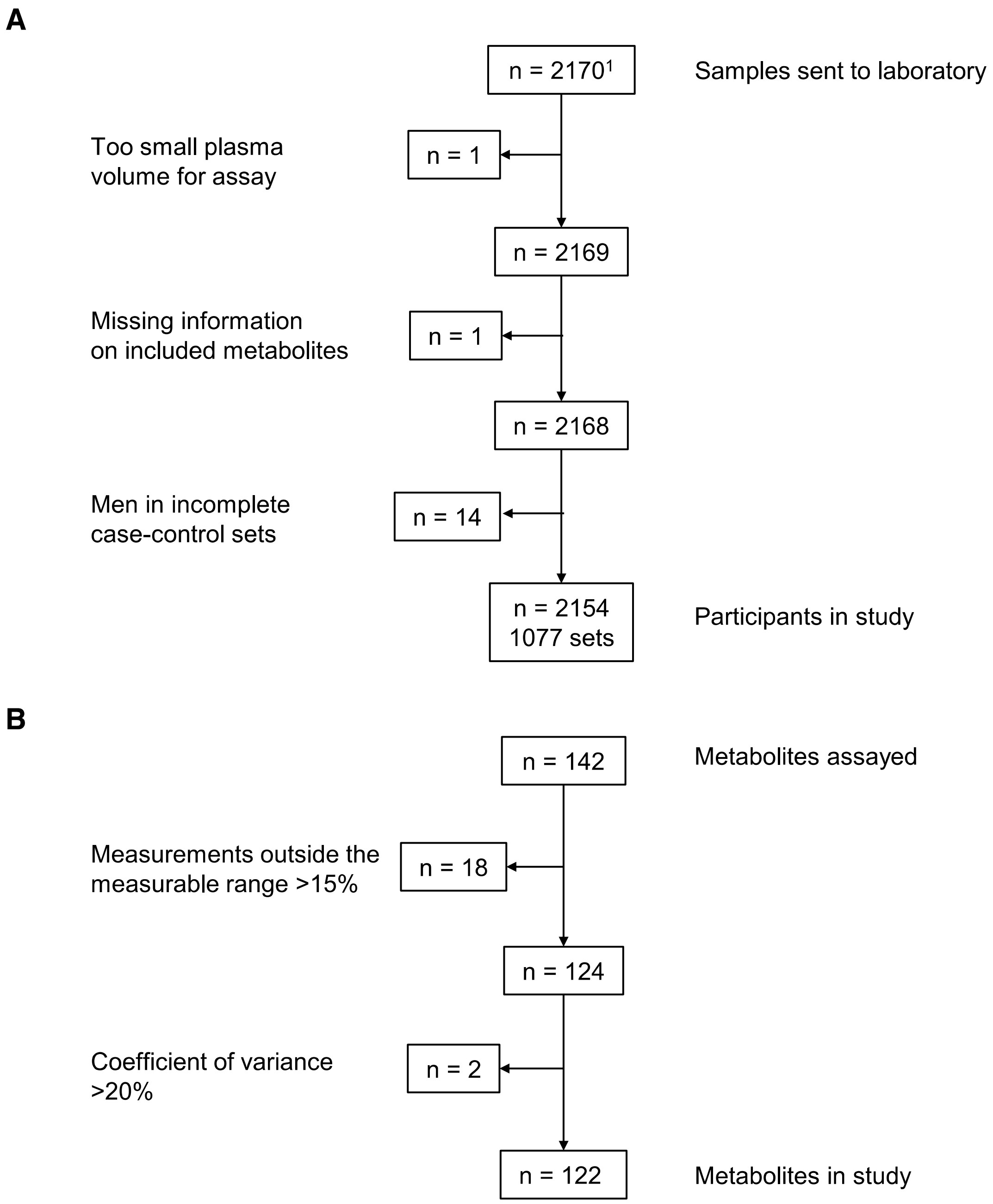


**Figure S1. Flowchart of exclusions of A: participants and B: metabolites.**

[1]There were 2162 samples but 8 samples contributed to the data set twice because three controls became cases and five controls were controls for two cases each.

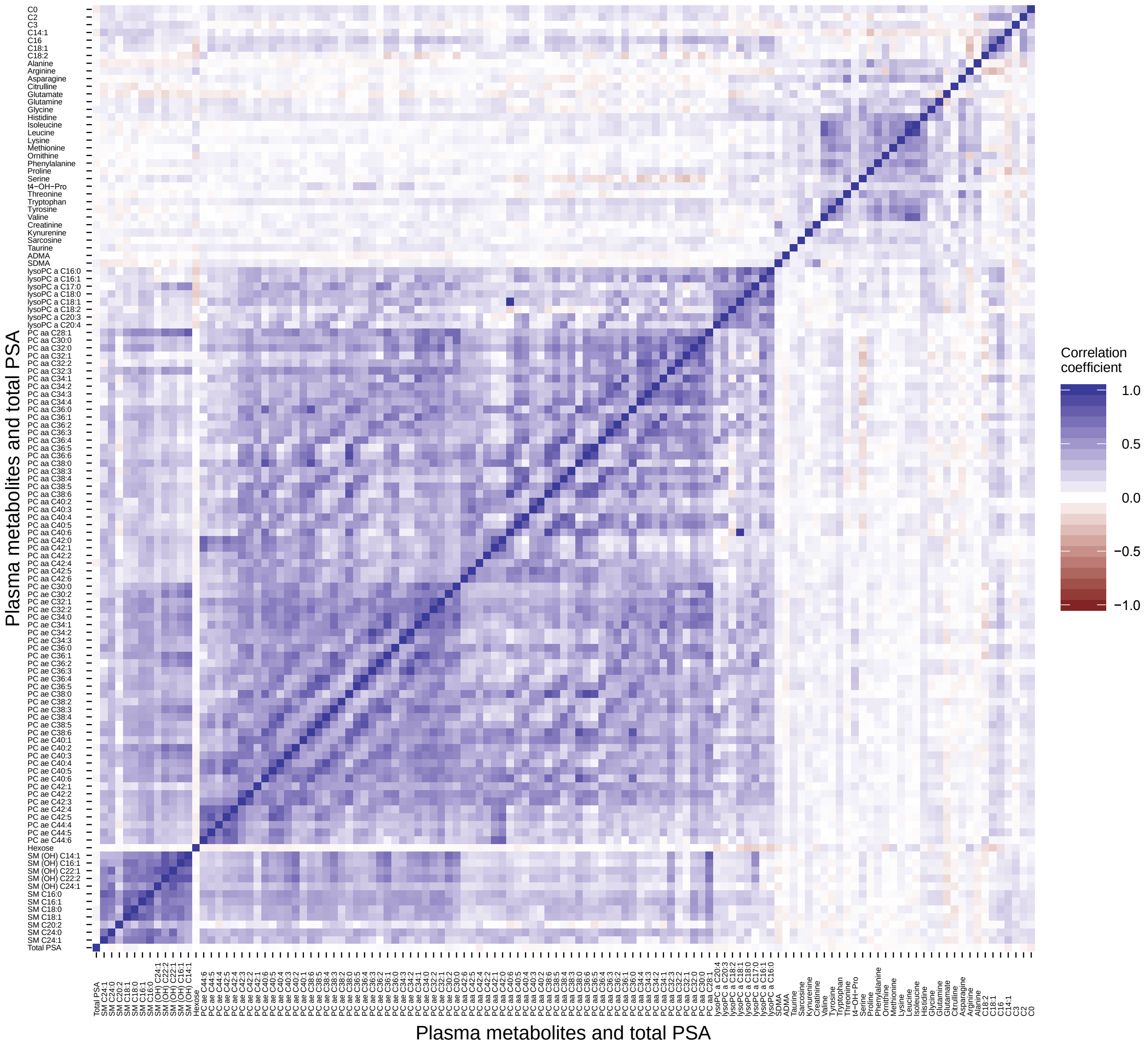


**Figure S2. Partial correlation coefficients between metabolite and total PSA concentrations at baseline.** Correlations between total PSA and metabolites were based on 764 controls for which total PSA was available, while correlations between metabolites included 1077 controls. The analyses were based on log-transformed data and were adjusted for age at blood collection (<55; 55-59; 60-64; 65-69; ≥70 years), body mass index (fourths; unknown) and study centre.

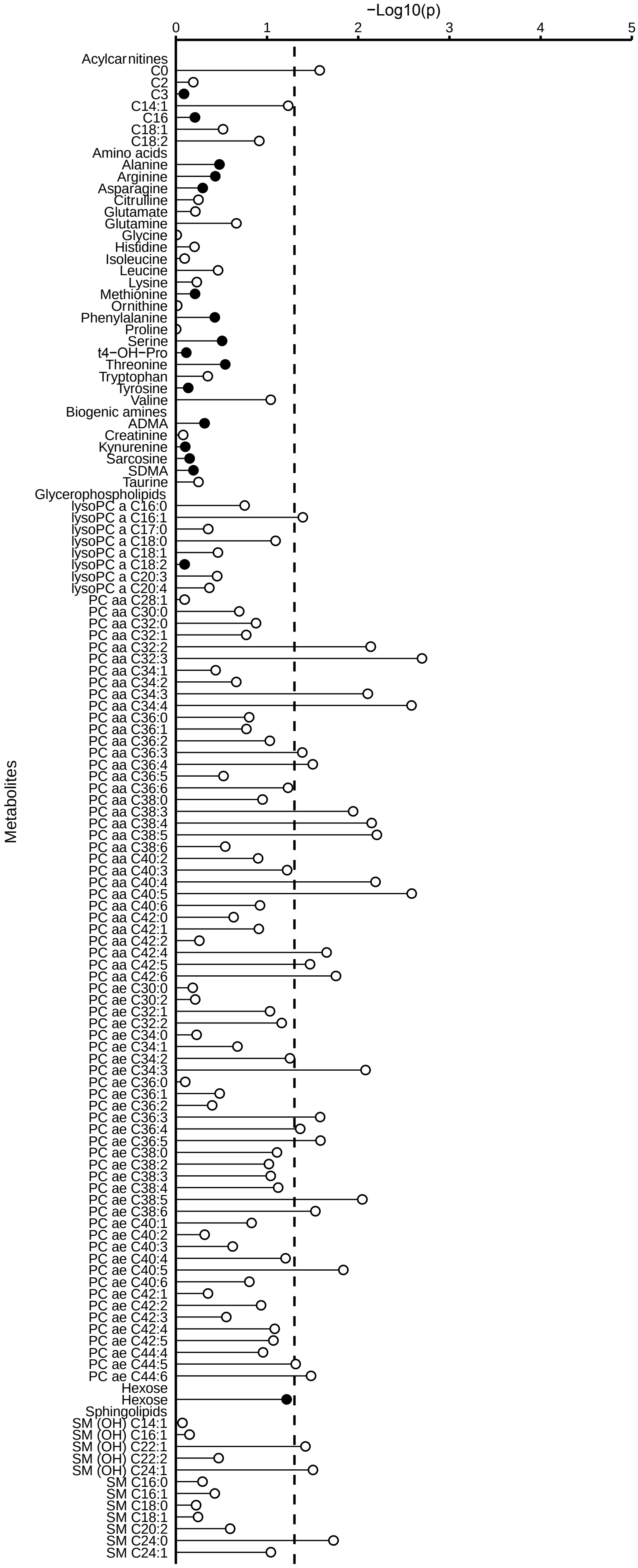


**Figure S3. Statistical significance of the associations between metabolite concentrations and risk of high grade prostate cancer.** High grade prostate cancer was tumours with Gleason score ≥8 or coded as undifferentiated tumours. The analysis included 124 matched case control sets. Statistical significance was plotted as –$\log_{10}$(p-values). The dashed line represents conventionally statistical significance at α = 0.05; no associations were statistical significance after controlling the false discovery rate at α = 0.05 (Benjamini-Hochberg). Filled circles represent positive associations and unfilled circles represent inverse associations. The p-values were derived from a conditional logistic regression using log-metabolite concentration as a continuous variable and adjusting for exact age (continuously), body mass index (fourths; unknown), smoking (never; past; current; unknown), alcohol intake (<10; 10-19; 20-39; ≥40 g of alcohol per day; unknown), education (primary or none; secondary; degree level; unknown) and marital status (married or cohabiting; not married or cohabiting; unknown).

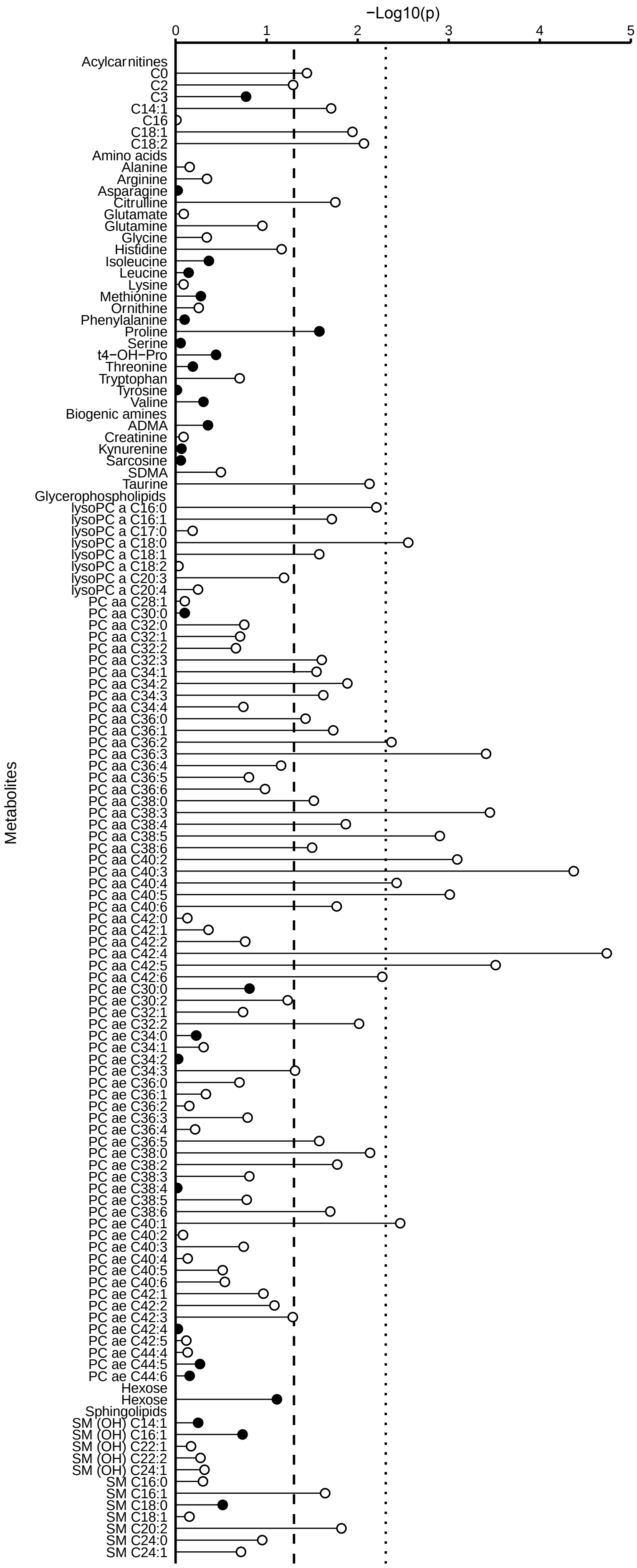


**Figure S4. Statistical significance of associations between metabolite concentrations and risk of advanced stage prostate cancer.** Advanced stage prostate cancer was tumours with TNM score of $T_{3-4}$ and/or $N_{1-3}$ and/or $M_1$, or coded as advanced. The analysis included 208 matched case control sets. Statistical significance was plotted as $-\log_{10}$(p-values). The dashed and the dotted lines represent conventionally statistical significance and statistical significance after allowing for multiple testing using a false discovery rate controlling procedure (Benjamini-Hochberg), respectively, both at α = 0.05. Filled circles represent positive associations and unfilled circles represent inverse associations. The p-values were derived from a conditional logistic regression using log-metabolite concentration as a continuous variable and adjusting for exact age (continuously), body mass index (fourths; unknown), smoking (never; past; current; unknown), alcohol intake (<10; 10-19; 20-39; ≥40 g of alcohol per day; unknown), education (primary or none; secondary; degree level; unknown) and marital status (married or cohabiting; not married or cohabiting; unknown).

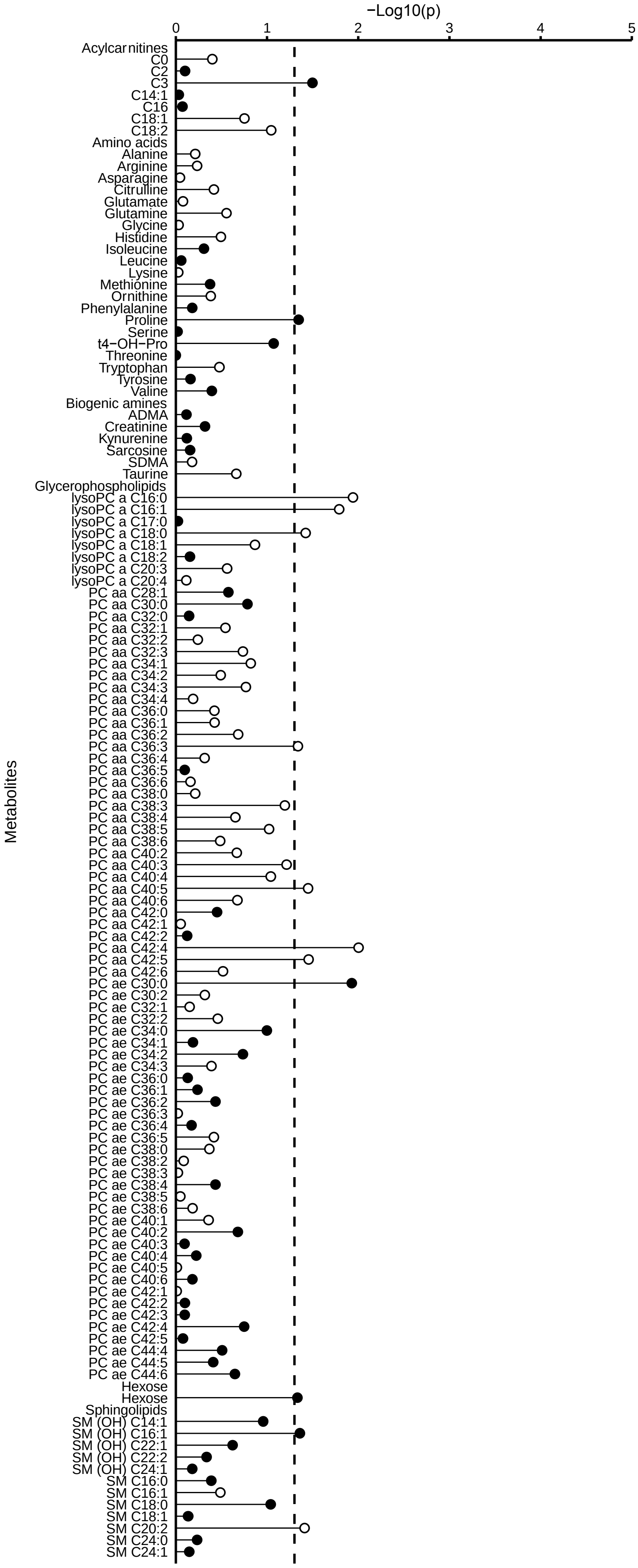


**Figure S5. Statistical significance of the associations between metabolite concentrations and risk of aggressive prostate cancer.** Aggressive prostate cancer was tumours with TNM score of $T_4$ and/or $N_{1\text{-}3}$ and/or $M_1$. The analysis included 115 matched case control sets. Statistical significance was plotted as $-\log_{10}$(p-values). The dashed line represents conventionally statistical significance at $\alpha = 0.05$; no associations were statistical significance after controlling the false discovery rate at $\alpha = 0.05$ (Benjamini-Hochberg). Filled circles represent positive associations and unfilled circles represent inverse associations. The p-values were derived from a conditional logistic regression using log-metabolite concentration as a continuous variable and adjusting for exact age (continuously), body mass index (fourths; unknown), smoking (never; past; current; unknown), alcohol intake (<10; 10-19; 20-39; ≥40 g of alcohol per day; unknown), education (primary or none; secondary; degree level; unknown) and marital status (married or cohabiting; not married or cohabiting; unknown).

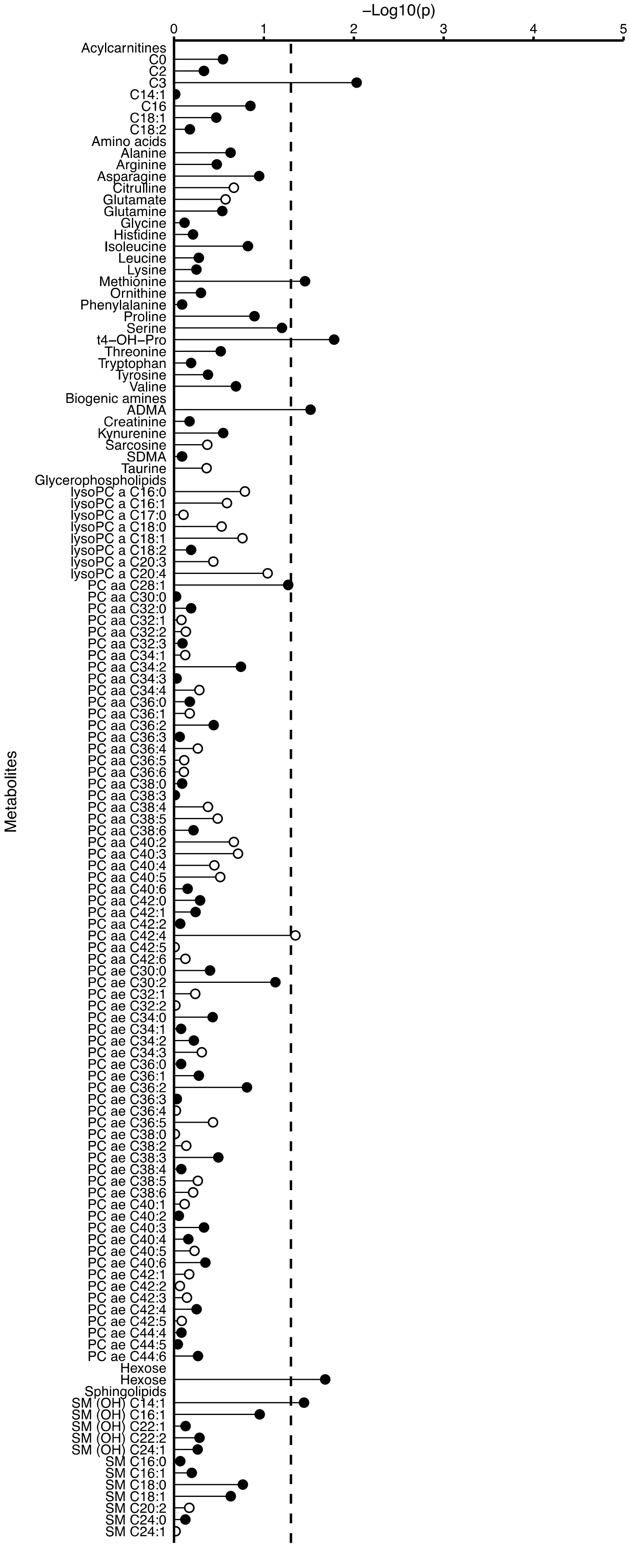


**Figure S6. Statistical significance of associations between metabolite concentrations and risk of death from prostate cancer.** The analysis included 127 matched case control sets. Statistical significance was plotted as $-\log_{10}$(p-values). The dashed line represents conventionally statistical significance at $\alpha = 0.05$; no associations were statistical significance after controlling the false discovery rate at $\alpha = 0.05$ (Benjamini-Hochberg). Filled circles represent positive associations and unfilled circles represent inverse associations. The p-values were derived from a conditional logistic regression using log-metabolite concentration as a continuous variable and adjusting for exact age (continuously), body mass index (fourths; unknown), smoking (never; past; current; unknown), alcohol intake (<10; 10-19; 20-39; ≥40 g of alcohol per day; unknown), education (primary or none; secondary; degree level; unknown) and marital status (married or cohabiting; not married or cohabiting; unknown).
